# Exploring the Neuroprotective Properties of Capsanthin: Antioxidant Defense and Inflammatory Responses

**DOI:** 10.3390/nu18010018

**Published:** 2025-12-19

**Authors:** Ramóna Pap, Edina Pandur, Gergely Jánosa, Adrienn Horváth, Kitti Tamási, Katalin Sipos, Attila Agócs, József Deli

**Affiliations:** 1 Department of Pharmaceutical Biology, Faculty of Pharmacy, University of Pécs, Rókus u. 2, H-7624 Pécs, Hungary; pandur.edina@gytk.pte.hu (E.P.); janosa.gergely@gytk.pte.hu (G.J.); horvath.adrienn2@pte.hu (A.H.); tamasi.kitti@gytk.pte.hu (K.T.); sipos.katalin@gytk.pte.hu (K.S.); 2 Department of Biochemistry and Medical Chemistry, Medical School, University of Pécs, Szigeti út 12, H-7624 Pécs, Hungary; attila.agocs@aok.pte.hu (A.A.); deli.jozsef@gytk.pte.hu (J.D.); 3 Department of Pharmacognosy, Faculty of Pharmacy, University of Pécs, Rókus u. 2, H-7624 Pécs, Hungary

**Keywords:** capsanthin, glutamate, oxidative stress, inflammation, antioxidants, neuron

## Abstract

Background/Objectives: Capsanthin is a xanthophyll carotenoid from Capsicum species with an extended conjugated polyene chain that underlies both its orange–red color and strong antioxidant potential. In this study, we investigated whether capsanthin protects RA-differentiated SH-SY5Y neuron-like cells against glutamate-induced stress. Methods: Neuronal dysfunction was induced by glutamate exposure, and capsanthin treatment was evaluated using cell viability, reactive oxygen species (ROS) production, antioxidant defense markers, inflammatory cytokines, mitochondrial energy status, and apoptosis-related endpoints. Antioxidant responses were assessed using superoxide dismutase, catalase, glutathione peroxidase activities, and total antioxidant capacity. Cytokine release (TNFα, IL-6, IL-8, IL-4, IL-10) was quantified by ELISA. Mitochondrial function was monitored using ATP content. Apoptosis-associated genes (BAX, BCL-2, CASP3, and CASP9) were analyzed using SYBR Green-based RT-qPCR, complemented by caspase-9 ELISA and caspase-3 Western blotting. Results: Glutamate increased oxidative stress and shifted the cytokine profile toward a pro-inflammatory state, accompanied by reduced ATP levels and a pro-apoptotic transcriptional pattern. Capsanthin significantly attenuated glutamate-induced ROS production, stabilized antioxidant enzyme activities and total antioxidant capacity, reduced pro-inflammatory cytokines while supporting anti-inflammatory signaling, and preserved ATP levels. Conclusions: Overall, capsanthin mitigated excitotoxic stress by maintaining redox balance, limiting inflammatory responses, and protecting mitochondrial energy metabolism in neuron-like cells, supporting its potential as a neuroprotective candidate for glutamate-induced neuronal stress.

## 1. Introduction

Carotenoids are natural lipid-soluble isoprenoid pigments found in plants, algae, fungi, and some bacteria, and they possess well-documented antioxidant and anti-inflammatory properties [1]. Beyond reactive oxygen species (ROS) scavenging, they are able to modulate membrane properties andredox-sensitive signaling pathways that influence inflammation and cell survival [2,3]. These features are relevant to neurodegenerative conditions, where oxidative stress and neuroinflammatory signaling often converge with excitotoxic mechanisms driven by glutamate [4].

Glutamate is the major excitatory neurotransmitter in the mammalian central nervous system, and it is essential for synaptic transmission, plasticity and memory [5]. However, in excess, it becomes neurotoxic (excitotoxicity) and is implicated in Alzheimer’s disease, Parkinson’s disease, amyotrophic lateral sclerosis, epilepsy, and stroke [6,7,8,9,10]. Overactivation of glutamate receptors results in intracellular Ca^2+^ overload, mitochondrial dysfunction, and increased ROS generation, which collectively promote inflammatory signaling and trigger apoptotic pathways [5,8].

Capsanthin is the main red xanthophyll in Capsicum species. Its extended conjugated polyene chain supports visible-light absorption, contributing to the orange–red color, while the conjugated π-electron system and oxygen-containing functional groups provide efficient energy dissipation and radical-quenching capacity, promoting strong antioxidant activity [11,12]. Consistent with these structural properties, capsanthin has been shown to exert photoprotective, anti-inflammatory, antidiabetic, chemopreventive, and anti-tumoral effects, which are likely related to ROS suppression, membrane biophysics, cellular transport, and modulation of inflammatory mediators [11,12,13,14,15,16,17,18,19,20,21]. Building upon capsanthin’s established anti-inflammatory and antioxidant capabilities, we hypothesized that it exerts neuroprotective effects, particularly against glutamate-mediated excitotoxicity, a critical mechanism underlying neurodegeneration. Capsanthin’s unique physicochemical features, are particularly relevant to the excitotoxic cascade which properties suggest it could interrupt several nodes of this cascade such as reinforcing endogenous antioxidant defenses (e.g., modulating SOD, CAT, GPx activity, and total antioxidant capacity) and nudging cytokine balance toward an anti-inflammatory profile, capsanthin may protect neurons from glutamate-initiated damage and limit the progression toward apoptosis [21].

Despite this background, mechanistic evidence for capsanthin in neuronal excitotoxic stress remains limited. In particular, it is still unclear whether capsanthin can simultaneously modulate antioxidant defenses, neuronal cytokine responses, and mitochondrial integrity in a glutamate injury model, an integrated profile that is critical for interpreting neuroprotective potential. Addressing this gap is important because excitotoxicity involves tightly coupled redox–inflammatory–mitochondrial processes rather than a single isolated pathway.

Retinoic acid (RA)-differentiated human SH-SY5Y neuroblastoma cells are widely used as a dopaminergic-like neuronal model for investigating excitotoxic mechanisms and screening neuroprotective interventions [22,23,24,25]. Therefore, this study aimed to clarify the potential antioxidant and anti-inflammatory effects of capsanthin, both alone and in a model of glutamate-induced neuronal cell damage. Using RA-differentiated glutamate-treated SH-SY5Y neurons, we examined changes in ROS and antioxidant protection, characterized changes in key cytokines across pro- and anti-inflammatory axes, and evaluated mitochondrial and apoptosis-related markers. By integrating these findings, we aimed to determine whether capsanthin can alleviate excitotoxic stress and map the putative mechanistic nodes through which protection occurs.

## 2. Materials and Methods

### 2.1. Cell Culture and Treatments

In our experiments, the SH-SY5Y human neuroblastoma cell line (CRL-2266; ATCC) was differentiated into dopaminergic neurons using retinoic acid. SH-SY5Y cells were maintained in cell culture medium Dulbecco’s Modified Eagle Medium/Nutrient Mixture F12 (DMEM F12; #DMEM-12-A; Lot.: CP24-7492; Capricorn Scientific GmbH, Ebsdorfergrund, Germany) containing 10% Fetal Bovine Serum (FBS; #ECS5000L; EuroClone S.p.A, Pero, Italy), 1% non-essential amino acids (NEAA; #NEAA-B; Lot.: CP23-6513; Capricorn Scientific GmbH, Ebsdorfergrund, Germany), and 1% penicillin/streptomycin (P/S; #PS-B; Lot.: CP24-7398; Capricorn Scientific GmbH, Ebsdorfergrund, Germany) at 37 °C in a 5% CO_2_ incubator. The cells were maintained, handled, and passaged under sterile conditions. Passage was performed when the cells reached 80% confluence. Trypsin (0.25% trypsin dissolved in PBS) was used to collect the cells. Sterile phosphate-buffered saline (PBS; Dulbecco’s phosphate-buffered saline; #PBS-1A; Capricorn Scientific GmbH, Ebsdorfergrund, Germany) was used to wash the cells. During passaging, cells were centrifuged at 130× *g* for 7 min, and after the treatments, cells were collected and centrifuged at 4580× *g* for 5 min. After centrifugation, the supernatant was aspirated, and the pellet was resuspended in a cell culture medium. Cell counting was performed using the Trypan Blue dye exclusion method with a Bürker cell counting chamber and Trypan Blue solution (0.4% and 0.85% in NaCl, #15250061; Gibco, Thermo Fisher Scientific Inc., Waltham, MA, USA).

SH-SY5Y cells were differentiated into dopaminergic neurons using 10 µM retinoic acid (RA; #R2526; Merck Life Science Kft., Budapest, Hungary) for 5 days [26,27]. For differentiation, RA was dissolved in absolute ethanol to a stock solution of 3 mg/mL, which was stored at −20 °C in a foil tube and used for up to 3 weeks. During the treatments, the amount of RA stock solution required for differentiation was diluted 10-fold in absolute ethanol, then retinoic acid was added to the differentiation medium (DMEM F12, 1% FBS, 1% NEAA) at the appropriate concentration (1 µL to 1 mL medium ratio), then added to the cells and differentiated in the incubator. Representative phase-contrast images of undifferentiated and 5-day RA-differentiated SH-SY5Y cells are provided in Supplementary Figure S4. Images were acquired using an EVOS XL Core microscope (Invitrogen, Carlsbad, CA, USA) with a Plan PH2 20×/0.40 objective.

After differentiation, the RA-containing medium was removed, and the cells were washed with PBS and treated with low-serum-containing cell culture medium. The cells were then treated with the same low-serum medium used for differentiation, without RA. During the experiments, RA-differentiated neurons were treated with capsanthin and/or glutamate. For capsanthin treatment, a stock solution of 1 mg/mL, freshly dissolved in DMSO and filtered for sterility, was used, and neurons were treated with different concentrations. As a control treatment, the same amount of DMSO as that of capsanthin was used. Untreated cells were used as absolute controls. A stock solution of 100 mM glutamate was prepared in DMSO and diluted using a cell culture medium, according to the cell treatment protocol. The dilutions were freshly prepared before treatment. Neurons treated with both capsanthin and glutamate received the treatments almost simultaneously, first capsanthin and then glutamate. In the preliminary glutamate experiments, the cells were treated with 1, 2, 3, 4, 5, 6, 7, 8, 9, 10, 15, and 20 mM glutamate. In the capsanthin preliminary experiments, the cells were treated with 5, 7, or 10 ng/µL capsanthin. Glutamate was used in concentrations of 1 mM and 5 mM for the treatments. Capsanthin was used in 10 ng/µL concentration for the treatments. Treatments were carried out at incubation intervals of 24 and 48 h. After treatment, the cells were stored in a 37 °C incubator containing 5% CO_2_. After incubation, the treated cells were collected, and 2 mL of the supernatant was also collected. Depending on the measurements, the cells were freshly used or stored at −20 °C until use, and the supernatants were kept at −80 °C.

### 2.2. Isolation of Capsanthin

The flesh of ripe red paprika (*Capsicum annuum* var. *longum nigrum*) was cut or blundered into small pieces and extracted with methanol twice overnight and once overnight with diethyl ether, each time using a tenfold amount of solvent. The methanolic fractions were evaporated and combined with the etherial fraction. To this solution, one-sixth of the amount of 30% methanolic KOH was added and kept overnight to complete the saponification. The ethereal phase was then washed with water 5–6 times to remove methanol and KOH from the organic phase. After drying and evaporation, the extract was partitioned between equal amounts of hexane (epiphase) and methanol/water 9:1 (hypophase). The separated hypophase contained polar carotenoids, including capsanthin. Toluene was added to the hypophase, where the carotenoids were transferred. The toluene phase was washed several times with water, dried, evaporated, and crystallized from toluene/hexane. The crystals contained > 60% capsanthin. The crystalline hypophasic extract was then subjected to silica gel chromatography using hexane/acetone (8:2 to 6:4) as the eluent. The fractions containing capsanthin were evaporated, and after crystallization, capsanthin was obtained with a purity of 85–98% purity. If the purity was below 95%, further chromatography on a calcium carbonate column (eluent toluene/hexane 8:2) could provide capsanthin with >98% purity as a red powder. All spectroscopic data were consistent with the literature data [28]. The above purities were assessed by HPLC measurements as described in [27] using a Teknochroma 250 × 4 mm C18-column: the eluent was 12% (*v*/*v*) H_2_O in methanol (A), methanol (B), 50% (*v*/*v*) acetone in methanol (C). The gradient program was 100% A for 8 min, to 80% A/20% B in 8 min, to 50% A/50% B in 8 min, to 100% B in 7 min, 100% B 2 min, to 100% C in 6 min, 100% C 5 min (linear steps). The flow rate was 1.5 mL/min. The peaks in a chromatogram were identified by means of authentic carotenoid samples and the UV−Vis spectra of the individual peaks. Photodiode array measurements of spectral properties for the individual peaks (from 300 to 510 nm) were determined at the upslope, apex, and downslope. The matching of the three spectra indicated the degree of peak purity. UV-Vis: λmax 476 nm (ethanol), m.p. 171–172 °C. 1H-NMR (500 MHz, CDCl3): δ = 0.84 (s, 3H, 16′-Me), 1.07 (s, 6H, 16, 17-Me), 1.20 (s, 3H, 17′-Me), 1.36 (s, 3H, 18′-Me), 1.45–1.51 (m, 2H, H-2ax, H-4′β), 1.73 (s, 3H, 18-Me), 1.76–1.79 (m, 2H, H-2eq, H-2′β), 1.95, 1.97, 1.99 (3 s, 12H, 19, 19′, 20, 20′-Me), 2.01 (m, 1H, H-2′α), 2.06 (m, 1H, H-4ax), 2.39 (dd, 1H, J = 5.4 Hz, J = 16.7 Hz, H-4eq), 2.95 (dd, 1H, J = 8.6 Hz, J = 14.5 Hz, H-4′α), 4.00 (m, 1H, H-3), 4.51 (m, 1H, H-3′), 6.12 (s, 2H, H-7, H-8), 6.16 (d, 1H, J = 11.6 Hz, H-10), 6.26 (d, 1H, J = 11.3 Hz, H-14), 6.35 (d, 1H, J = 10.0 Hz, H-14′), 6.36 (d, 1H, J = 15.4 Hz, H-12), 6.44 (d, 1H, J = 15.0 Hz, H-7′), 6.51 (d, 1H, J = 14.6 Hz, H-12′), 6.55 (d, 1H, J = 11.3 Hz, H-10′), 6.58–6.73 (m, 4H, H-11, 11′, 15, 15′), 7.32 (d, 1H, J = 15.0 Hz, H-8′).

13C-NMR (125 MHz, CDCl3): δ = 12.8–12.9 (4C, C-19, C-19′, C-20, C-20′), 21.4 (C-18′), 21.6 (C-18), 25.2 (C-17′), 26.0 (C-16′), 28.8 (C-16), 30.3 (C-17), 42.7 (C-4), 44.0 (C-1′), 45.5 (C-4′), 48.6 (C-2), 51.0 (C-2′), 59.0 (C-5′), 65.1 (C-3), 70.4 (C-3′), 121.0 (C-7′), 124.1 (C-11′), 125.6 (C-11), 125.9 (C-7), 126.3 (C-5), 129.7 (C-15′), 131.3 (C-10), 131.7 (C-15′), 132.4 (C-14), 133.7 (C-9′), 135.2 (C-14′), 135.9 (C-13′), 137.5 (C-12), 137.6 (C-13), 137.9 (C-6), 138.5 (C-8), 140.6 (C-10′), 142.0 (C-12′), 146.9 (C-8′), 202.8 (C-6′). The HPLC chromatogram at 450 nm with peak integration and the UV–Vis spectrum of the main peak are presented in Supplementary Figure S8.

### 2.3. Viability Measurements

Resazurin-based in vitro toxicity (#TOX8-1KT; Lot.: 0000338679; Merck Life Science Kft., Budapest, Hungary) measurements were used to determine the viability of the cells. For the measurements, 10,000 cells/well were differentiated with retinoic acid (RA) for five days. After differentiation and medium exchange, the cells were treated with different concentrations of capsanthin (5–7–10 ng/µL) and glutamate (1–10 mM, 15 mM, and 20 mM) and incubated for 6, 24, 48, and 72 h. Subsequently, viability measurements were conducted. Resazurin staining solution (20 µL in 100 µL cell suspension) was pipetted to the cells, then the 96-well plates were incubated for 2 h at 37 °C. After incubation, the optical density was detected colorimetrically at 600 nm using a Multiskan GO spectrophotometer (Thermo Scientific Inc., Waltham, MA, USA). Supplementary Figures S1–S3 present the results obtained from different concentrations of capsanthin and glutamate treatments with their controls. Raw OD values for all viability experiments are provided in Supplementary Table S1 (Excel file).

### 2.4. Reactive Oxygen Species Measurements

A fluorometric assay, the Cellular Reactive Oxygen Species Detection Assay Kit (Deep Red Fluorescence) (#AB186029; Lot.: 1086977-3; Abcam, Cambridge, UK), was used to detect intracellular ROS in living cells. Measurements were performed in quadruplicate according to the manufacturer’s instructions. For the measurement, SH-SY5Y cells were differentiated with RA for 5 days (10,000 cells/well) and, after medium exchange, treated with 1 mM or 5 mM glutamate and 10 ng/µL of capsanthin. In the reactions, ROS Deep Red solution was prepared and added to the cells, which were then incubated at 37 °C for 10-20-30-40-50-60 min. After incubation, the plates were measured using a Perkin Elmer Multimode fluorescence plate reader in the 650 excitation and 675 nm emission range in the bottom read mode. After 40 min of incubation, no differences in the ROS levels were observed. Therefore, our results are plotted up to the 30 min measurements. The fluorescence intensities obtained for the ROS variations were compared with those of the control groups. Data are expressed as the ratio of relative fluorescence intensity (RFI). Supplementary Figure S4 presents the results obtained from different concentrations of capsanthin and glutamate treatments with their controls.

### 2.5. Enzyme-Linked Immunosorbent Assay Measurements

RA-differentiated cells were treated as previously described. After the treatments, the cell culture medium was collected and stored at −80 °C until measurements were performed. Anti-human IL-6 (#E0090Hu; BT LAB, Bioassay Technology Laboratory, Zhejiang, China), IL-4 (#BMS225-2), IL-8 (#KHC0081), IL-10 (#KHC0101), and TNFα ELISA kits (#BMS223-4; Lot.: 427721-007; Invitrogen, Thermo Fisher Scientific Inc., Waltham, MA, USA) were used to measure the levels of the secreted cytokines. Measurements were performed according to the manufacturer’s protocol. The absorbance was measured using a Multiskan GO spectrophotometer (Thermo Fisher Scientific Inc., Waltham, MA, USA) at 450 nm. The measured optical density was directly proportional to the concentration of the target cytokines in the original samples. The concentrations of the target cytokines were calculated using SkanIt RE 5.0 software compared to the standards and presented as pg/mL.

Caspase-9 levels were determined according to the manufacturer’s protocol using a Human Caspase 9 ELISA Kit (#BMS2025; Lot.: 422488-006; Invitrogen, Thermo Fisher Scientific Inc., Waltham, MA, USA). Briefly, freshly collected cells were lysed at a concentration of 5 × 10^6^ cells/mL and incubated at room temperature with gentle shaking for 60 min. Samples were centrifuged at 1000× *g* for 15 min, and the purified lysate was used for testing. The measurements were performed on 96-well plates. The plates were washed twice with PBS. The standards were diluted according to the manufacturer’s protocol. The samples were diluted twice. After adding the detection antibody, the plates were incubated for 2 h. The plates were then washed three times, and the HRP conjugate was added for the reactions and incubated for 1 h. The washing steps were repeated, and TMB substrate was added to the wells and incubated in the dark for 10 min. Absorbance was measured at 450 nm using a Multiskan GO spectrophotometer (Thermo Fisher Scientific Inc.) after adding a stop solution. The concentration of caspase-9 was calculated based on a standard curve using an analysis program and is expressed in ng/mL.

### 2.6. Antioxidant Capacity Measurements

Antioxidant Assay Kit (#MAK334-1KT; Lot.: 334CE11A25; Merck Life Science Kft., Budapest, Hungary) was used to determine the total antioxidant capacity of the cells. Measurements were performed in triplicate according to the manufacturer’s instructions. For the measurements, 10^6^ cells were treated and collected for each reaction. Briefly, 20 µL of samples and Trolox standards were pipetted into the wells, 100 µL of the reaction mixture was added, and the 96-well plates were incubated for 10 min at room temperature. Antioxidants reduce Cu^2+^ to Cu^+^, which forms a color complex with the reagent. The optical density of the samples was measured using a Multiskan GO spectrophotometer (Thermo Fisher Scientific Inc., Waltham, MA, USA) at 570 nm, where the intensity of the color was proportional to the antioxidant capacity. Antioxidant capacity was expressed in µM.

### 2.7. Superoxide Dismutase Activity Measurements

The Superoxide Dismutase Activity Assay Kit (#MAK528-1KT; Lot.: 528CE05A24; Merck Life Science Kft., Budapest, Hungary) was used to determine SOD enzyme activity in the cells. The cells were treated and collected as described above. For each reaction, 10^6^/dish freshly collected cells were used. Measurements were performed in triplicate according to the manufacturer’s instructions. Briefly, freshly collected cells were lysed in cold lysis buffer (0.1M Trizma-HCl containing 0.5% Triton X-100 and 5 mM mercaptoethanol) and protease inhibitors and centrifuged at 14,000× *g* for 5 min. The supernatants were used in the reactions. Next, 20 µL of samples and standards were added to the wells, and 160 µL of WST working reagent was added to each reaction, followed by the addition of 20 µL of xanthine oxidase working reagent. The plates were then incubated at room temperature for 30 min. The absorbance was measured at 450 nm using a Multiskan GO spectrophotometer (Thermo Fisher Scientific Inc., Waltham, MA, USA). Superoxide is generated by xanthine oxidase, which reacts with WST dye. SOD inhibits this process. The degree of color loss was proportional to the enzyme activity. SOD activity was expressed in U/mL.

### 2.8. Catalase Measurements

Catalase levels in the cells were determined using a Catalase Assay Kit (#MAK531-1KT; Lot.: 531CF07A15; Merck Life Science Kft., Budapest, Hungary). The cells were treated as described above. After treatment, 10^6^ cells/reaction were collected and used for measurements, which were performed in triplicate. The cells were centrifuged at 1000× *g* for 10 min at 4 °C, and the pellet was homogenized on ice in a 50 mM potassium phosphate buffer containing 1 mM EDTA. The lysate was centrifuged at 10,000× *g* for 15 min at 4 °C, and the supernatant was used for the measurements. Briefly, 12 µL of 1 mM H_2_O_2_ was added to each well to initiate the reactions and incubated for 30 min at 25 °C. Next, 10 µL of stop solution was added to the wells, followed by the addition of 50 µL developer reaction mix. The plates were incubated for 10 min at 25 °C. The unconverted H_2_O_2_ produced a chromogenic product, which was measured at 540 nm using a Multiskan GO spectrophotometer (Thermo Fisher Scientific Inc., Waltham, MA, USA). Enzyme activity was calculated using the catalase activity equation, where one unit refers to the amount of catalase enzyme that decomposes 1 µmol of H_2_O_2_ per minute at pH 4.5 and 25 °C (in U/L).

### 2.9. ATP Measurements

An ATP Assay Kit (#MAK190; Merck Life Science Kft., Budapest, Hungary) was used to determine ATP levels in the cells. The cells were treated as described above. Freshly collected cells were used for the reactions at a density of 10^6^ cells per reaction. The measurements were performed in triplicate according to the manufacturer’s protocol. The samples were added to the wells to a final volume of 50 µL, and 50 µL of the reaction mixture was added to each well. The plates were incubated for 30 min at room temperature, protected from the light. The ATP content of the cells was quantified using spectrophotometry at 570 nm. ATP concentration was calculated according to the protocol equation. The results were expressed as ng/µL.

### 2.10. Glutathione Peroxidase Activity Measurements

GPx measurements (#MAK437-1KT; Lot.: 437CE09A09; Merck Life Science Kft., Budapest, Hungary) were performed using a spectrophotometric method based on the utilization of NADPH. After treatment, 10^6^ cells were freshly collected and used for the reactions. Measurements were performed in triplicate according to the manufacturer’s protocol. GPx catalyzes the reduction of H_2_O_2_ by converting glutathione to its oxidized form. The resulting GSSG is reduced to GSH by the enzyme glutathione reductase (GR) using NADPH. NADPH extinction was measured at 340 nm using a Multiskan GO spectrophotometer (Thermo Fisher Scientific Inc., Waltham, MA, USA). The results were expressed in units per liter (U/L).

### 2.11. RNA Isolation

The RNA isolation was performed using the Bio-Rad Aurum™ Total RNA Mini Kit (#7326820; Bio-Rad Inc., Hercules, CA, USA) according to the manufacturer’s protocol. After treatment, the cells were collected using a cell scraper and washed with PBS. Isolation was performed from 500000 cells per treatment. First, 350 μL of lysis buffer was added to the cells, followed by 350 μL of ethanol, and the samples were suspended and pipetted into the RNA binding columns. After centrifugation for 30 s at 15,000× *g*, the flow-through was discarded, and the RNA samples were washed with 700 μL of low-stringency wash buffer. Next, DNase digestion was performed for 15 min at room temperature. The RNA-binding column was washed with 700 μL of high-stringency buffer for 30 s, followed by 700 μL of low-stringency buffer for 1 min. The tubes were centrifuged for 2 min, and the samples were eluted with the elution buffer. The RNA concentration was measured using a Multiskan GO spectrophotometer and analyzed using SkanIt software (Thermo Fisher Scientific Inc., Waltham, MA, USA). The isolated RNA samples were stored at −20 °C until further use.

### 2.12. cDNA Synthesis

Isolated total RNA was used to generate complementary DNA (cDNA) using the iScript cDNA Synthesis Kit (#1708897; Bio-Rad Inc., Hercules, CA, USA) according to the manufacturer’s protocol. For each synthesis, 200 ng of total RNA was used for each reaction. The RNA was pipetted into 0.5 mL PCR tubes, made up to 15 μL with nuclease-free water, and placed on ice. The Master Mix reaction mixture contained 1 µL/reaction of iScript Reverse Transcriptase enzyme and 4 µL/reaction of 5x iScript Select Reaction Mix. The 5 μL of Master Mix per reaction was prepared and mixed with the 15 μL RNA sample to obtain a final reaction volume of 20 μL. After pipetting the Reaction Mix to the RNA samples, the tubes were centrifuged, and cDNA synthesis was performed using an Eppendorf Mastercycler^®^ personal thermal cycler (Eppendorf Austria GmbH, Wien, Austria). The synthesized cDNA samples were stored at −20 °C until further use.

### 2.13. qReal-Time PCR Measurements

The expression of apoptosis regulators was measured using SYBR Green-based (#1725121; Bio-Rad Inc., Hercules, CA, USA) quantitative real-time PCR. Beta-actin was used as the housekeeping control gene. The expression changes were determined using the ΔΔCt-method with Bio-Rad CFX Maestro 3.1 software. The primer sequences used in the qRT-PCR are listed in Table 1.

### 2.14. Western Blot Measurements

For Western blot analysis, 10^6^ cells/dish were treated and collected as described above. The samples were lysed in cold lysis buffer using an ultrasonic sonicator for 2 min, then centrifuged at 183× *g* at 4 °C for 5 min. The protein concentrations were measured using a detergent-compatible protein assay kit (DC Kit, #5000111; Bio-Rad Inc., Hercules, CA, USA). Then, protein samples were separated by sodium dodecyl sulfate gel electrophoresis (SDS-PAGE) at 200 V using a Mini-PROTEAN Tetra Cell system (Bio-Rad Inc., Hercules, CA, USA). The proteins were then transferred to nitrocellulose membranes using a Trans-Blot Turbo transfer system (Bio-Rad Inc., Hercules, CA, USA). EveryBlot blocking buffer (#12010020; Merck Life Science Kft., Budapest, Hungary) was used to block the membranes. An anti-human cleaved caspase-3 antibody (1:3000 in BSA; #AB3623; Merck Life Science Kft., Budapest, Hungary) for primary protein labelling and rabbit anti-human glyceraldehyde-3-phosphate dehydrogenase (GAPDH; 1:3000 in fat-free milk powder; #G9545; Merck Life Science Kft., Budapest, Hungary) antibody as a housekeeping protein. Goat anti-rabbit IgG antibody (1:12000; #AP307P; Merck Life Science Kft., Budapest, Hungary) was used as the secondary antibody. The membranes were incubated overnight at 4 °C with the caspase-3 antibody. Chemiluminescent detection was performed using WesternBright ECL substrate (#K-12045-D50; Advansta Inc., San Jose, CA, USA). An Alliance Q9 gel documentation system (UVITec, Cambridge, UK) was used for imaging. NineAlliance Q9 Uvitec software v18.16c software was used for data analysis. The expression of the target protein was calculated as the percentage of the target/housekeeping protein ratio.

### 2.15. Statistics

The presented data are representative of independent experiments, where *n* refers to the number of independent experiments. Within each experiment, reaction measurements were performed in technical triplicate. The number of independent experiments was as follows: cell viability (*n* = 5), ROS (*n* = 5), catalase activity (*n* = 3), SOD activity (*n* = 3), GPx activity (*n* = 3), ELISA measurements for cytokines (*n* = 3), qRT-PCR (*n* = 3), caspase-3 Western blot (*n* = 3), caspase-9 ELISA (*n* = 4), and ATP measurements (*n* = 4).

Statistical analyses were performed using IBM SPSS Statistics for Windows version 26 (IBM Corporation, Armonk, NY, USA). The significance was assessed using one-way analysis of variance (ANOVA) with LSD post hoc tests. Data are presented as mean values in the graphs, with error bars indicating standard deviations (SDs).

The level of significance was set at *p* < 0.05. Significant differences compared to the own controls are indicated by the * symbol, and significant changes compared to glutamate-treated cells are indicated by the † symbol.

## 3. Results

### 3.1. Cell Viability

First, viability measurements were performed to determine the appropriate concentration for the experiment. The preliminary results of all the concentration measurements are presented in Supplementary Figures S1–S3.

**Figure 1:**
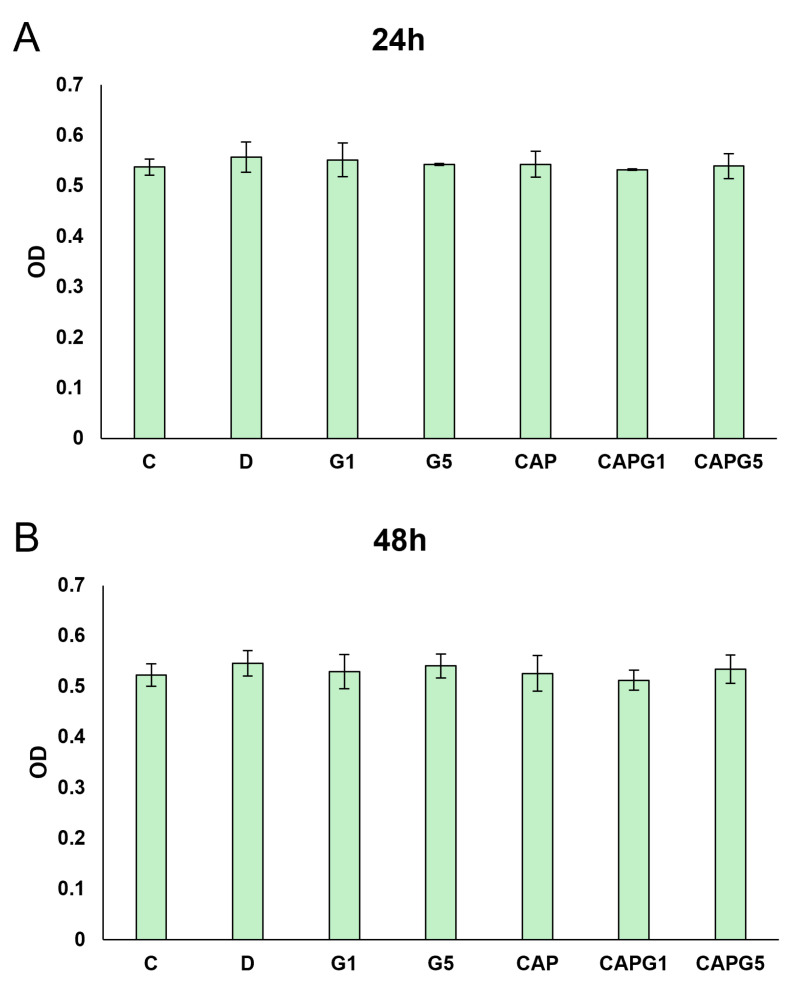
The viability of cells after 24 h (**A**) and 48 h (**B**) capsanthin and glutamate treatment.

Our results showed that treatment with glutamate or capsanthin alone or in combination with glutamate did not affect cell viability (Figure 1).

### 3.2. Oxidative Stress and Antioxidant Defense

As oxidative stress is the primary mechanism of glutamate-induced cell death, next, we measured ROS levels. The preliminary results of each concentration of ROS measurements are presented in Supplementary Figure S4. Antioxidant capacity is the primary indicator that specifies the ability of cells to neutralize free radicals and assesses the potential role of oxidative stress in diseases. For this reason, the total antioxidant capacity (TAC) of the cells was measured. Oxidative stress triggers modulations in the activity of antioxidant enzymes; therefore, the activities of superoxide dismutase (SOD), glutathione peroxidase (GPx), and catalase (CAT) were also investigated.

**Figure 2:**
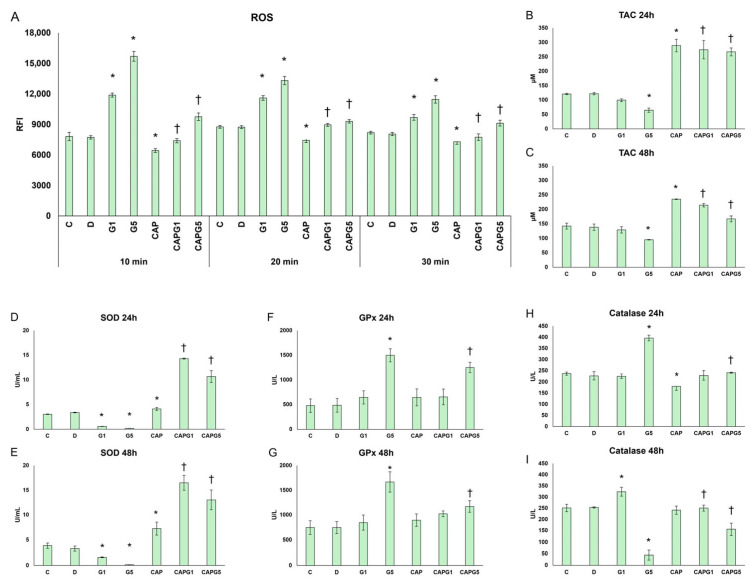
Redox and antioxidant changes in cells after capsanthin and glutamate treatment. (**A**) Levels of Reactive Oxygen Species in cells after capsanthin and glutamate treatment (*n* = 5). (**B**,**C**) Antioxidant capacity in cells after 24 h (**B**) and 48 h (**C**) of capsanthin and glutamate treatment (*n* = 3). (**D**,**E**) Superoxide dismutase activity in cells after 24 h (**D**) and 48 h (**E**) of capsanthin and glutamate treatment (*n* = 3). (**F**,**G**) Glutathione peroxidase activity in cells after 24 h (**F**) and 48 h (**G**) of capsanthin and glutamate treatment (*n* = 3). (**H**,**I**) Catalase activity in cells after 24 h (**H**) and 48 h (**I**) of capsanthin and glutamate treatment (*n* = 3). Significant differences (*p* < 0.05) compared to the controls are indicated by the * symbol, and significant changes (*p* < 0.05) compared to glutamate-treated cells are indicated by the † symbol. Abbreviations: C—absolute control; D—DMSO control; G1—1 mM glutamate; G5—5 mM glutamate; CAP—capsanthin; CAPG1—capsanthin and 1 mM glutamate; CAPG5—capsanthin and 5 mM glutamate.

According to our results, glutamate strongly increased ROS levels. The presence of capsanthin significantly reduced ROS levels in the presence of glutamate, suggesting an antioxidant effect (Figure 2A).

Treatment with 1 mM glutamate moderately decreased the antioxidant capacity of cells, while 5 mM glutamate significantly decreased the antioxidant capacity of cells, suggesting the stress-related depletion of antioxidants.

Capsanthin treatment elevated the antioxidant capacity after 24 h as well as 48 h, and in the presence of glutamate, it was able to normalize or increase the antioxidant capacity, suggesting its protective role in the cells (Figure 2B,C).

Next, the SOD enzyme activity was determined, as it plays a role in the primary defense against oxidative stress, preventing the direct damaging effects of superoxide and its disruptive impact on cell signaling. In our experiments, both concentrations of glutamate treatment decreased significantly SOD activity in the RA-differentiated cells. Capsanthin induced a mild increase, while in the presence of glutamate, it further elevated the SOD activity in the cells. This suggests that capsanthin treatment reduces superoxide production, thereby secondarily reducing the burden on SOD, which may increase its enzyme activity (Figure 2D,E).

After SOD, the glutathione peroxidase activity was measured. Treatment with 5 mM glutamate significantly increased GPx activity after both 24 h and 48 h experiments. Capsanthin alone did not induce an alteration in the enzyme activity. Furthermore, capsanthin modulated GPx activity in cells treated with capsanthin and glutamate. The increase observed with 5 mM glutamate may be a compensatory response to the increased peroxide load, and capsanthin may normalize this effect by reducing peroxide production (Figure 2F,G).

In our 24 h treatments, 5 mM glutamate increased catalase activity in response to stress. After 48 h, 1 mM glutamate induced a delayed, adaptive increase in catalase activity in response to prolonged but moderate stress. Upon 5 mM glutamate treatment, a strong decrease in enzyme activity was observed, suggesting severe enzyme inactivation and/or dysfunction. Capsanthin alone decreased slightly catalase activity after 24 h, suggesting a lower enzyme requirement, while it returned to physiological levels after 48 h. Capsanthin in the presence of 1 mM glutamate did not alter the enzyme activity, while together with 5 mM glutamate, the catalase peak triggered by G5 was normalized, suggesting a protective effect after 24 h. In addition, partial recovery was observed after 48 h (Figure 2H,I). These results are consistent with the TAC reduction and severe oxidative depletion.

Overall, glutamate exposure induced significant oxidative stress, characterized by increased ROS production, disruption of antioxidant enzyme balance (decreased SOD and stress-induced changes in GPx and CAT), and a concentration- and time-dependent decrease in the total antioxidant capacity. Capsanthin alone slightly enhanced the cellular antioxidant status without affecting the viability. During the combined treatment, capsanthin consistently reduced ROS production, prevented glutamate-induced depletion or inactivation of antioxidant enzymes, and restored TAC to levels near or above the control values. These results suggest that capsanthin enhances the redox defense system of neuronal cells and compensates for glutamate-induced oxidative damage in neuronal cells.

### 3.3. Inflammatory Responses

**Figure 3:**
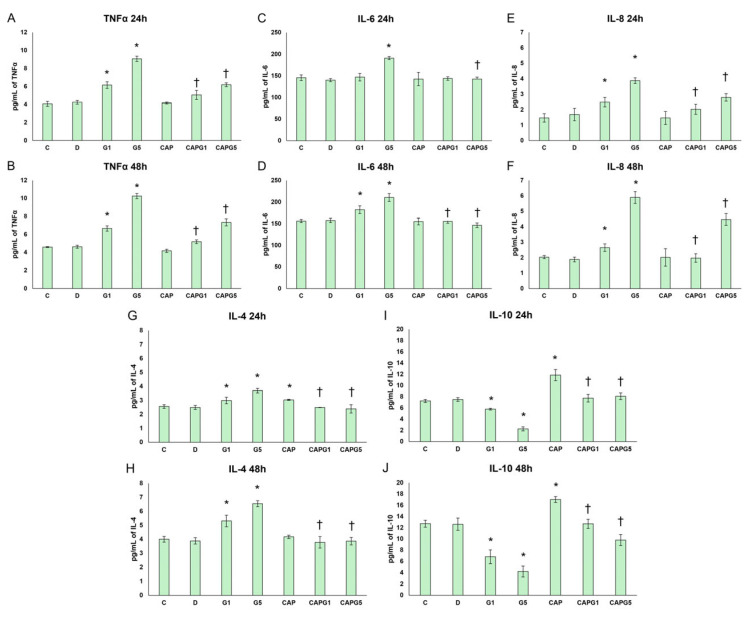
Inflammatory changes in cells after capsanthin and glutamate treatments. (**A**,**B**) TNF-α secretion after 24 h (**A**) and 48 h (**B**) of capsanthin and glutamate treatment (*n* = 3). (**C**,**D**) IL-6 secretion after 24 h (**C**) and 48 h (**D**) of capsanthin and glutamate treatment (*n* = 3). (**E**,**F**) IL-8 secretion after 24 h (**E**) and 48 h (**F**) of capsanthin and glutamate treatment (*n* = 3). (**G**,**H**) IL-4 secretion after 24 h (**G**) and 48 h (**H**) of capsanthin and glutamate treatment (*n* = 3). (**I**,**J**) IL-10 secretion after 24 h (**I**) and 48 h (**J**) of capsanthin and glutamate treatment (*n* = 3). Significant differences (*p* < 0.05) compared to the controls are indicated by the * symbol, and significant changes (*p* < 0.05) compared to glutamate-treated cells are indicated by the † symbol. Abbreviations: C—absolute control; D—DMSO control; G1—1 mM glutamate; G5—5 mM glutamate; CAP—capsanthin; CAPG1—capsanthin and 1 mM glutamate; CAPG5—capsanthin and 5 mM glutamate.

Oxidative stress triggers inflammatory responses. Therefore, the next step in our experiments was to monitor cytokine levels. First, the proinflammatory cytokine TNF-α levels were measured. Following glutamate treatment, TNF-α secretion increased in a concentration- and time-dependent manner. Capsanthin in the presence of glutamate (CAPG1, CAPG5) significantly decreased the TNF-α levels compared to glutamate treatments (Figure 3A,B).

Next, we investigated the dual role of IL-6. The secretion of IL-6 increased after glutamate treatment in a concentration- and time-dependent manner, suggesting a damaging neuroinflammatory pattern. Capsanthin alone did not modify the IL-6 levels, but in the presence of glutamate (CAPG1, CAPG5), capsanthin normalized IL-6 secretions to physiological levels (Figure 3C,D).

In the central nervous system, the main sources of IL-8 are microglia and astrocytes, although neurons can also produce IL-8 in response to oxidative stress and inflammatory stimuli. Therefore, the secretion of the CXC chemokine IL-8 was also monitored. Glutamate treatment induced IL-8 secretion by neurons, whereas capsanthin, in the presence of glutamate (CAPG1 and CAPG5), reduced or normalized this effect (Figure 3E,F).

Therefore, we were also interested in changes in anti-inflammatory cytokines and examined the secretion of IL-4 and IL-10.

In glutamate treatments, IL-4 secretion increased, with greater glutamate leading to higher IL-4 levels, especially at 48 h, suggesting an anti-inflammatory neuronal supportive signal for these cells. Capsanthin alone caused a mild protective priming with a slight increase in IL-4 at 24 h, which evens out at 48 h experiments. This decrease in IL-4 levels alongside capsanthin probably indicates that less anti-inflammatory compensation is needed because the proinflammatory effect is lower. In the combined treatments (CAPG1 and CAPG5), capsanthin significantly reduced IL-4 at both time points compared to the corresponding glutamate, which was approximately at the control levels (Figure 3G,H).

Next, we determined the secretion of the anti-inflammatory key regulator cytokine IL-10. IL-10 levels were significantly reduced by glutamate treatment, consistent with the predominance of proinflammatory activity. Capsanthin alone could increase the IL-10 secretion of the cells, while in the presence of glutamate, capsanthin modulated the IL-10 levels to normal. These results suggest that capsanthin has an IL-10-restoring effect, possibly improving cell protection (Figure 3I,J).

Taken together, glutamate induced a strong proinflammatory profile in RA-differentiated SH-SY5Y cells, with concentration- and time-dependent increases in TNF-α, IL-6, and IL-8 levels, along with a simultaneous decrease in the crucial anti-inflammatory cytokine IL-10 and a reactive increase in IL-4. Capsanthin alone exerted a mild anti-inflammatory effect, manifested in a slight increase in IL-4 and IL-10, without altering the proinflammatory mediators. Under glutamate-induced stress, capsanthin significantly reduced the secretion of TNF-α, IL-6, and IL-8 and normalized IL-4 and IL-10 levels, thus shifting the cytokine environment from a detrimental proinflammatory state to a more balanced neuroprotective profile.

### 3.4. Apoptosis

The outcome of oxidative and inflammatory stress is often cell death or apoptosis. To determine this process, first, BAX, BCL-2, pro-CASP3 and CASP9 qRT-PCR measurements were performed.

In the 5 mM glutamate-treated cells, the expression of apoptosis-promoting genes BAX, CASP3, and CASP9 increased after 24 h, while the anti-apoptotic gene BCL-2 decreased. This indicates the activation of early apoptotic signal transduction in response to oxidative stress.

In cells treated with capsanthin and glutamate, the expression ratio decreased significantly, the BAX/BCL-2 ratio was normalized, and CASP3 and CASP9 partially returned to control levels. This suggests capsanthin-mediated cell protection and inhibition of apoptosis.

After 48 h, the expression of apoptotic genes further increased upon 5 mM glutamate treatment, especially CASP3 and CASP9, whereas BCL-2 expression decreased even more, indicating cell death.

**Figure 4:**
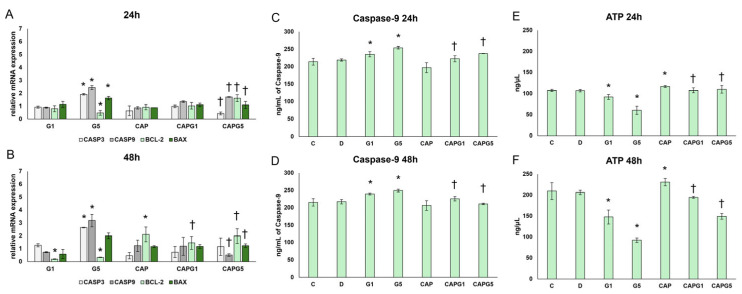
Apoptotic changes in cells after capsanthin and glutamate treatment. (**A**,**B**) Relative mRNA expression of CASP3, CASP9, BCL-2, and BAX genes after 24 h (**A**) and 48 h (**B**) of capsanthin and glutamate treatment (*n* = 3). (**C**,**D**) The caspase-9 protein levels after 24 h (**C**) and 48 h (**D**) of capsanthin and glutamate treatment (*n* = 4). (**E**,**F**) ATP levels after 24 h (**E**) and 48 h (**F**) of capsanthin and glutamate treatment (*n* = 4). Significant differences (*p* < 0.05) compared to the own controls are indicated by the * symbol, and significant changes (*p* < 0.05) compared to glutamate-treated cells are indicated by the † symbol. Abbreviations: C—absolute control; D—DMSO control; G1—1 mM glutamate; G5—5 mM glutamate; CAP—capsanthin; CAPG1—capsanthin and 1 mM glutamate; CAPG5—capsanthin and 5 mM glutamate.

Capsanthin in the presence of glutamate decreased the expression of the examined pro-apoptotic genes, whereas that of BCL-2 was relatively restored. This suggests a lasting anti-apoptotic and mitochondrial membrane-stabilizing effect, accompanied by a decrease in ROS levels and normalization of catalase activity (Figure 4A,B).

Next, protein experiments were performed to verify whether the observed changes in gene expression were reflected at the translational level of the protein. For this purpose, caspase-3 Western blotting and caspase-9 ELISA were performed.

In the case of caspase-9, increased levels were detected after 24 h and 48 h of glutamate treatment. Capsanthin alone did not induce changes in caspase-9 expression. In cells treated with capsanthin and glutamate, a normalization was observed in caspase-9 levels (Figure 4C,D).

In parallel, cleaved caspase-3 was assessed by Western blot; however, no significant changes were detected at either time point, and these data are presented in Supplementary Figure S6. The combination of increased caspase-9 but unchanged cleaved caspase-3 suggests that mitochondrial apoptotic signalling was initiated but did not proceed to full execution under these conditions, potentially reflecting an incomplete caspase cascade and/or a sublethal stress state constrained by endogenous inhibitory mechanisms and cellular energy availability.

Given the central role of mitochondria in excitotoxic injury, intracellular ATP content was next measured to evaluate bioenergetic status. After 24 h, glutamate reduced ATP levels in a concentration-dependent manner, consistent with early mitochondrial dysfunction. Capsanthin alone slightly increased ATP content, and co-treatment effectively prevented glutamate-induced ATP depletion. After 48 h, ATP levels declined further in the 5 mM glutamate group, indicating more pronounced mitochondrial impairment, whereas capsanthin maintained significantly higher ATP levels in glutamate-treated cells, supporting preservation of mitochondrial energy metabolism and cellular viability (Figure 4E,F).

Our results showed the oxidative stress and mitochondrial damage-inducing effects of glutamate, which were indicated by the increased ROS and caspase-9 protein levels with decreased ATP levels in the cells, suggesting the initiation of intrinsic apoptosis. The absence of an increase in cleaved caspase 3-protein suggests that the apoptosis is not proceeding, which is probably due to metabolic collapse or partial activation of compensatory survival mechanisms.

Capsanthin treatment significantly alleviated these disorders by reducing oxidative stress, inhibiting the apoptotic process, stabilizing the function of the antioxidant enzymes studied, and maintaining mitochondrial ATP synthesis.

Overall, our results highlighted that capsanthin can preserve the physiological function of RA-differentiated neuronal cells by maintaining antioxidant responses against oxidative stress, preserving mitochondrial integrity, and preventing the progression of apoptosis, thus supporting its potential as a natural compound that protects against glutamate-induced neuronal stress.

## 4. Discussion

In our study, we analyzed glutamate-induced oxidative and inflammatory stress, mitochondrial damage, and intrinsic apoptotic pathways in RA-differentiated SH-SY5Y neuronal cells as well as the extent to which capsanthin can modify these processes. Overall, as expected, glutamate induced concentration- and time-dependent oxidative stress, antioxidant system disruption, proinflammatory cytokine profile, mitochondrial dysfunction, and early apoptotic signal activation, whereas capsanthin largely alleviated or normalized all of these, without significantly reducing cell viability.

Glutamate excitotoxicity is now a well-known key mechanism in neurodegenerative diseases, and excessive glutamate loading leads to increased ROS formation, depletion of antioxidant defenses, mitochondrial dysfunction, and ultimately, apoptotic or necrotic cell death [8,29,30,31]. In our study, glutamate significantly increased ROS levels while decreasing the total antioxidant capacity of cells in a dose-dependent manner, particularly at a concentration of 5 mM. This is consistent with in vitro data showing that glutamate increases intracellular ROS production, reduces the efficiency of the antioxidant system, and damages mitochondria in both SH-SY5Y cells and other neuronal models [32,33,34,35]. However, viability did not decrease significantly in the presence of either glutamate or capsanthin after the 24–48 h time interval. This suggests that the chosen glutamate concentrations and treatment times targeted an early stage of the excitotoxic cascade, where cells already showed clear signs of oxidative and inflammatory stress, but cell death had not yet occurred or had only partially occurred. This period of reversible stress is particularly important for studying neuroprotective effects, as it models situations where intervention can reverse the process or mitigate the extent of damage.

The changes observed in SOD, GPx, and catalase activities form a complex but easily interpretable pattern. Glutamate significantly reduced SOD activity at both concentrations, while GPx activity increased in response to 5 mM glutamate, and catalase showed a time- and dose-dependent pattern, initially compensatory, suggesting depletion at 5 mM after 48 h. This scenario fits well with the idea that a persistently high ROS load initially triggers increased enzyme activity (especially at the level of GPx and catalase), but that oxidative modification of enzyme proteins and depletion of antioxidant cofactors (e.g., glutathione) lead to a failure of antioxidant protection in the longer term [36,37,38].

However, in the presence of capsanthin, SOD activity increased and was partially normalized in the presence of glutamate, GPx activity decreased compared to the overcompensation induced by 5 mM glutamate, and catalase approached control values, especially under higher glutamate load. These results suggest that capsanthin reduces superoxide and peroxide stress through its direct ROS-scavenging properties, while delicately regulating intracellular redox homeostasis and preventing excessive activation and subsequent depletion of antioxidant enzymes [16,39]. This effect may be due to its configuration, which is based on its characteristic orange-red color and high antioxidant capacity, achieved by promoting energy distribution and electron delocalization in the extended π system [39,40].

In the case of total antioxidant capacity, the findings can be summarized as glutamate caused a concentration-dependent decrease, capsanthin alone caused a moderate increase, and in combination with glutamate, normalization or an increase was observed. These results are consistent with the neuroprotective, mitochondrial-stabilizing, and ROS-reducing effects of other carotenoids, such as astaxanthin and fucoxanthin [41,42,43].

Oxidative stress and chronic inflammation are closely intertwined processes that reinforce one another and contribute to the progression of neurodegenerative diseases [37,44,45].

In our results, the levels of TNF-α, IL-6, and IL-8 increased in a concentration- and time-dependent manner in response to glutamate, while the level of IL-10, a key anti-inflammatory cytokine, decreased, and that of IL-4 increased reactively. This pattern can be described as a proinflammatory, neurotoxic milieu in which the increase in IL-4 can be interpreted as a self-protective, compensatory attempt by neurons. However, with a decrease in IL-10, this compensation is clearly insufficient [46,47,48,49].

Capsanthin alone did not significantly alter TNF-α, IL-6, and IL-8 levels, but it induced a slight increase in IL-4 and IL-10 levels and, in the presence of glutamate, significantly reduced the levels of proinflammatory cytokines (TNF-α, IL-6, IL-8) and normalized IL-4 and IL-10 concentrations. This profile clearly indicates an anti-inflammatory shift: cells experience glutamate-induced damage in a less destructive and more regeneration-supportive cytokine environment. This is consistent with the known anti-inflammatory effects of capsanthin and other pepper carotenoids: animal models and macrophage systems have shown that capsanthin reduces IL-6 and TNF-α production and generally moderates oxidative and inflammatory markers [14,18,50,51].

Our results suggest that in this model, capsanthin intervenes in the excitotoxic process at several points, as it reduces ROS formation and restores total antioxidant capacity, normalizes the activity of antioxidant enzymes (SOD, GPx, and catalase), shifts the proinflammatory cytokine environment toward a more balanced neuroprotective profile (TNF-α, IL-6, IL-8 decrease, and IL-10 and IL-4 normalization), and inhibits the progression of the intrinsic apoptotic pathway (BAX/BCL-2, CASP9, ATP). Our current findings are novel in that a similar anti-inflammatory pattern can be observed in an RA-differentiated neuronal model, even against a background of glutamate excitotoxicity.

Upon glutamate treatment, the levels of proapoptotic BAX, CASP3, and CASP9 mRNA expression increased at a concentration of 5 mM after 24 h, while anti-apoptotic BCL-2 decreased, and this pattern intensified further after 48 h. This clearly indicates the activation of the intrinsic apoptotic pathway. The protein levels of caspase-9 also increased, whereas cleaved caspase-3 protein levels did not alter significantly at any time point.

This apparent difference, the strong apoptotic gene expression and caspase-9 signaling, but unchanged cleaved caspase-3 levels and retained viability, can be interpreted as a process in which the apoptotic cascade is initiated, but mitochondrial energy metabolism breakdown and parallel survival mechanisms prevent complete caspase-dependent apoptosis. Glutamate excitotoxicity places mitochondria at the center of damage according Ca^2+^ overload, mitochondrial membrane potential destabilization, increased mitochondrial ROS generation, and permeability transition, collectively driving bioenergetic failure. Crucially, several steps of intrinsic apoptosis are energy-dependent, including apoptosome function; therefore, a decrease in ATP below a critical threshold can limit downstream execution despite upstream pro-apoptotic signaling. Accordingly, elevated caspase gene expression and increased caspase-9 levels can reflect initiation/priming of the pathway, while the absence of cleaved caspase-3 may indicate that full caspase execution is constrained by insufficient cellular energy charge and/or endogenous brakes on the caspase cascade [52,53,54,55]. This is supported by the change in ATP levels; we observed a concentration-dependent decrease in ATP in response to glutamate, which worsened over 48 h, indicating impaired mitochondrial function. ATP recovery should also be interpreted cautiously, because large ATP content integrates multiple processes beyond oxidative phosphorylation. A higher ATP signal can arise from preserved mitochondrial coupling and reduced ATP drain under stress, but it may also reflect compensatory metabolic reprogramming (e.g., increased glycolytic contribution) and differences in ATP consumption linked to inflammatory/oxidative burden. Therefore, to pinpoint the bioenergetic mechanism behind the ATP normalization, future work should combine ATP measurements with direct assessments of mitochondrial function (mitochondrial membrane potential assays, oxygen consumption and glycolytic flux measurements, and permeability transition–related readouts). Mitochondrial protection is inferred from ATP preservation, modulation of redox status, and caspase-9 signaling rather than from direct mitochondrial membrane potential measurements; and the lack of change in cleaved caspase-3 and overall viability suggests that apoptosis is not fully executed under these conditions, but definitive discrimination between reversible stress and progressing apoptosis will require future studies employing Annexin V/PI staining, mitochondrial membrane potential assays, and more detailed mitochondrial functional readouts [30,56].

In the presence of capsanthin, the BAX/BCL-2 ratio normalized, CASP3 and CASP9 mRNA levels partially returned to control values, and caspase-9 protein levels decreased, while ATP levels increased and were also compensated by glutamate.

This suggests a mitochondrial-stabilizing effect in which capsanthin, by reducing ROS levels, preserving the function of antioxidant enzymes, and moderating the inflammatory response, prevents irreversible loss of mitochondrial membrane potential and full activation of the caspase cascade. A similar pattern has been described for other carotenoids in models of traumatic or oxidative brain injury, in which antioxidant treatment reduced mitochondrial ROS, attenuated apoptosis, and improved cellular energy supply [42,57].

The RA-differentiated SH-SY5Y cell line is a widely used neuronal model, particularly for studying dopaminergic cells, and provides relevant information on the pathomechanism of neurodegenerative diseases [25,58,59,60].

During RA-induced differentiation, cells acquire a more mature neuronal phenotype, neurite formation, and increased synaptic marker expression, and they become more sensitive to various toxic stimuli, including glutamate exposure [61,62].

This multitarget effect is particularly promising in neurodegenerative diseases, where the disease process is shaped not by a single pathomechanism but by mutually reinforcing mechanisms (oxidative stress, inflammation, mitochondrial dysfunction, and excitotoxicity) [8,43].

Capsanthin, together with other carotenoids, has the potential to be used as a nutritional or pharmaconutritional intervention that can reduce neuronal stress and slow progression in the long term. However, further studies are needed to assess translational relevance, particularly regarding the pharmacokinetics of capsanthin, its brain penetration, and the feasibility of human dosing ranges [41,51]. Importantly, the concentration applied here was selected to model a maximally effective, non-cytotoxic exposure in vitro, but this does not constitute a pharmacokinetic justification. In cell culture, neither systemic absorption nor distribution can be captured, and the effective intracellular exposure is influenced by protein binding, cellular uptake, and compound stability. Human data show that capsanthin appears in plasma after paprika intake, yet its overall bioavailability is low, highlighting that achieving brain-relevant exposure in vivo is a non-trivial translational step. Therefore, the next logical stage is to test capsanthin in formulated delivery systems (e.g., lipid-based or nanoencapsulated carriers) and to perform dedicated in vivo pharmacokinetic and brain-penetration studies to determine whether biologically effective concentrations can be reached beyond the blood–brain barrier (BBB) [63,64,65].

### Limitations and Future Directions

Our study has some limitations that affect the generalizability and translational evaluation of the results. An RA-differentiated SH-SY5Y cell line was used, which, although widely accepted as a neuronal model, is tumor-derived and only partially reflects the complex properties of primary human neurons, even after differentiation [58,60].

In addition to neurons, astrocytes, microglia, oligodendrocytes, and endothelial cells are key players in the central nervous system. It is particularly important for the interpretation of the cytokine response that our model is purely neuronal in nature, such that changes in IL-6, IL-8, or TNF-α originate exclusively from neuronal sources and do not reflect the actual contribution of the glial component [66,67,68,69].

Therefore, the observed anti-inflammatory effects in our model are neuron-specific actions, and not as a full representation of the complex, multicellular inflammatory environment present in the brain with glia cells, which are the main mediators of neuroinflammation [69]. Future studies should include neuron-glia co-cultures or in vivo models where the bidirectional communication between neurons, astrocytes, and microglia can be examined. This would help determine whether the protective effects observed here extend to physiological conditions where glial cells are the dominant mediators of neuroinflammation.

In this study, only two glutamate concentrations and two incubation times were examined. This provides an insight into acute and early excitotoxic processes. However, chronic and disease-relevant exposure patterns, such as prolonged low-level glutamatergic stress, repeated insults, and delayed inflammatory and mitochondrial adaptations, cannot be modeled within the scope of the present experimental design. Therefore, the effects of chronic, low-dose glutamate exposure and the long-term protective efficacy of capsanthin remain to be established. Additionally, we did not test a wide range of capsanthin doses; therefore, the dose–response relationship and potential hormetic effects remain for the current data. Future studies should include extended exposure paradigms and broader dosing schemes, ideally complemented by neuron–glia co-culture systems or in vivo models to better reflect disease-relevant excitotoxic conditions.

In our experiments, we applied a single capsanthin concentration (10 ng/µL), although lower non-toxic doses were also identified in the preliminary viability assays. Our intention was to use the highest concentration that did not reduce cell viability, as we expected this dose to exert the most pronounced antioxidant and protective effects under glutamate-induced stress. Since our objective was not to map the entire dose–response curve, but to determine whether capsanthin can counteract oxidative and apoptotic processes under excitotoxic conditions, we chose to focus on the maximally effective non-cytotoxic concentration. Nevertheless, future studies should systematically explore the full concentration range to determine whether capsanthin exhibits hormetic behaviour or plateaus in its neuroprotective efficacy. Although animal data suggest that capsanthin has systemic antioxidant and immunomodulatory potential, further pharmacokinetic and clinical studies are required for human translation [14].

The translational relevance of the applied capsanthin concentration also requires careful consideration. The 10 ng/µL dose used in our in vitro assays corresponds to approximately 17 µM, which is higher than typical human plasma concentrations reported after dietary intake (0.05–2 µM) but falls within the range achievable by targeted or pharmacological supplementation [63,70]. The BBB presents an additional limitation, as carotenoids generally display low but measurable BBB permeability, and only a fraction of circulating capsanthin is expected to reach neural tissue under physiological conditions [71].

Consequently, while our results demonstrate clear neuroprotective effects at the cellular level, further pharmacokinetic studies are required to determine whether comparable concentrations can be achieved in the brain in vivo. These data will be essential for defining realistic dosing strategies, clarifying BBB transport efficiency, and establishing whether capsanthin or its metabolites could exert similar antioxidant and anti-inflammatory actions under physiological conditions.

Overall, our results showed that capsanthin can effectively reduce the early, oxidative, and inflammatory components of glutamate excitotoxicity in RA-differentiated SH-SY5Y neuronal cells, stabilize the antioxidant defense system, improve mitochondrial energy metabolism, and inhibit the progression of the intrinsic apoptotic pathway.

Although the conclusions should be treated with caution due to the limitations of the present study, capsanthin appears to be a promising candidate as part of a complex, multi-target neuroprotective strategy that may contribute to slowing the progression of neurodegenerative diseases characterized by glutamate excitotoxicity in the long term.

## 5. Conclusions

In summary, our results showed that capsanthin mitigated glutamate-induced excitotoxic stress in RA-differentiated SH-SY5Y neuron-like cells by reducing ROS production, stabilizing antioxidant defenses, and shifting the cytokine profile toward a less pro-inflammatory state. Capsanthin also preserved cellular ATP levels and attenuated intrinsic apoptotic signaling (BAX/BCL-2 balance and caspase-9), supporting a protective effect on mitochondrial integrity and energy metabolism. Although these findings are limited to a neuronal in vitro model, they provide mechanistic evidence that capsanthin may act as a neuroprotective candidate under glutamate-driven stress and justify further validation in neuron–glia systems and in vivo models, including pharmacokinetic and BBB assessment. In addition, the dose-selection strategy identified a non-cytotoxic capsanthin range in RA-differentiated neurons, providing a practical basis for subsequent mechanistic and translational studies. Future work should establish a protective dose–response relationship across the major endpoints and incorporate direct mitochondrial readouts (e.g., membrane potential and respiratory function) to better define the bioenergetic mechanism underlying ATP preservation. Finally, formulation-driven delivery approaches and brain distribution studies will be essential to determine whether capsanthin or its metabolites can reach neuroprotective concentrations in vivo.

## Figures and Tables

**Table 1:** Primer sequences used in the qRT-PCR reactions.

Primer Name	5′-3′ Sequence
forward Act	AGA AAA TCT GGC ACC ACA CC
reverse Act	GGG GTG TTG AAG GTG TCA AA
forward pro-CASP3	TTT CAG AGG GGA TCG TTG T
reverse pro-CASP3	CAC TGT CTG TCT CAA TGC C
forward CASP9	CCA GTG ACA TCT TTG TGT CC
reverse CASP9	GCA ACC AGG CAT CTG TTT AT
forward BAX	CCT TTT GCT TCA GGG TTT CA
reverse BAX	CTC CAT GTT ACT GTC CAG TTC
forward BCL-2	CCT TCT TTG AGT TCG GTG G
reverse BCL-2	GAG AAA TCA AAC AGA GGC CG

## Data Availability

Data is contained within the article and Supplementary Material.

## Supplementary Materials

The following supporting information can be downloaded at: https://www.mdpi.com/article/10.3390/nu18010018/s1, Figure S1: The viability of cells after 6 h (A), 24 h (B), 48 h (C), and 72 h (D) treatment with different concentrations of capsanthin (dissolved in DMSO) and the corresponding DMSO control.; Figure S2: The viability of cells after 6 h following treatment with different concentrations of glutamate (A) and the corresponding DMSO control (B); and after 24 h following treatment with different concentrations of glutamate (C) and the corresponding DMSO control (D). Figure S3: The viability of cells after 48 h following treatment with different concentrations of glutamate (A) and the corresponding DMSO control (B); and after 72 h following treatment with different concentrations of glutamate (C) and the corresponding DMSO control (D). Figure S4: Reactive Oxygen Species levels after 10 min (A), 20 min (B), and 30 min (C) of glutamate and its control treatments. Figure S5: The caspase-3 protein levels after 24 h (A,C) and 48 h (B,D) of capsanthin and glutamate treatment. Figure S6: Representative phase-contrast images of undifferentiated (A,B) and 5-day RA-differentiated (C,D) SH-SY5Y. Figure S7: Caspase-3 and GAPDH original Western blot images with sample and labels. Figure S8: (A) Analytical HPLC chromatogram recorded at 450 nm showing the major capsanthin peak and minor components. (B) Peak integration table with retention times and relative peak areas (%) used for purity estimation by peak area normalization. (C) UV–Vis spectrum of the main HPLC peak (capsanthin). Table S1. Raw OD values from cell viability assays (In Vitro Toxicology colorimetric assay, 600 nm). Data are provided as an Excel file.
