# Supplementary material for: Exploring the Neuroprotective Properties of Capsanthin: Antioxidant Defense and Inflammatory Responses
Source: Nutrients. 2025 Dec 19;18(1):18. doi: 10.3390/nu18010018 (PMC12787947; doi:10.3390/nu18010018)
Supplement: Supplementary file 1 [file nutrients-18-00018-s001.zip › nutrients-4017437-supplementary.pdf]

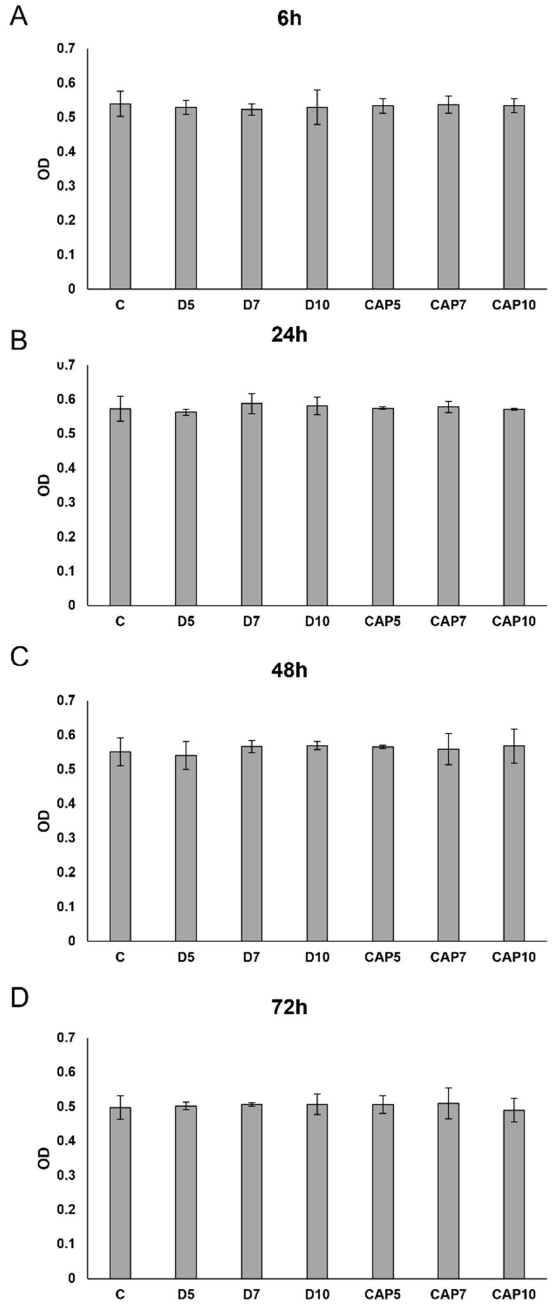

Supplementary Figure S1. The viability of cells after 6 h (**A**), 24 h (**B**), 48 h (**C**), and 72 h (**D**) treatment with different concentrations of capsanthin (dissolved in DMSO) and the corresponding DMSO control. Abbreviations: C-control; D5-5ng/μL capsanthin equal DMSO; D7-7ng/μL capsanthin equal DMSO; D10-10ng/μL capsanthin equal DMSO; CAP5-5ng/μL capsanthin; CAP7-7ng/μL capsanthin; CAP10-10ng/μL capsanthin; (*n*=5)

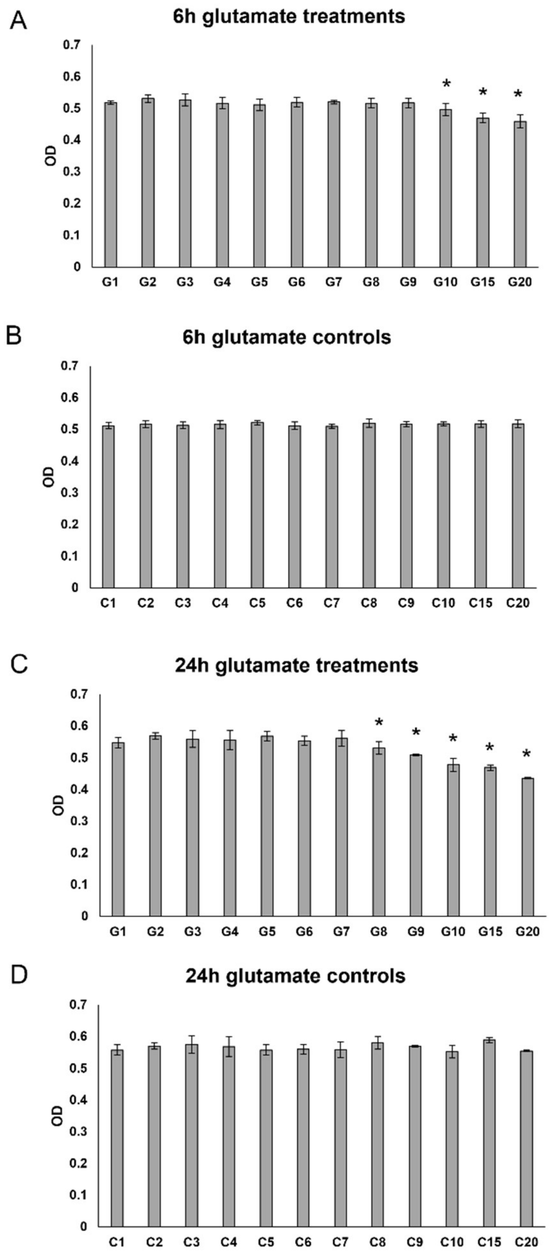

Supplementary Figure S2. The viability of cells after 6 h following treatment with different concentrations of glutamate (**A**) and the corresponding DMSO control (**B**); and after 24 h following treatment with different concentrations of glutamate (**C**) and the corresponding DMSO control (**D**). Significant differences ($p < 0.05$) compared to the DMSO controls are indicated by the * symbol. Abbreviations: G1-1mM glutamate; G2-2mM glutamate; G3-3mM glutamate; G4-4mM glutamate; G5-5mM glutamate; G6-6mM glutamate; G7-7mM glutamate; G8-8mM glutamate; G9-9mM glutamate; G10-10mM glutamate; G15-15mM glutamate; G20-20mM glutamate; C1-control of 1mM glutamate; C1-control of 1mM glutamate; C2-control of 2mM glutamate; C3-control of 3mM glutamate; C4-control of 4mM glutamate; C5-control of 5mM glutamate; C6-control of 6mM glutamate; C7-control of 7mM glutamate; C8-control of 8mM glutamate; C9-control of 9mM glutamate; C10-control of 10mM glutamate; C15-control of 15mM glutamate; C20-control of 20mM glutamate (n=5).

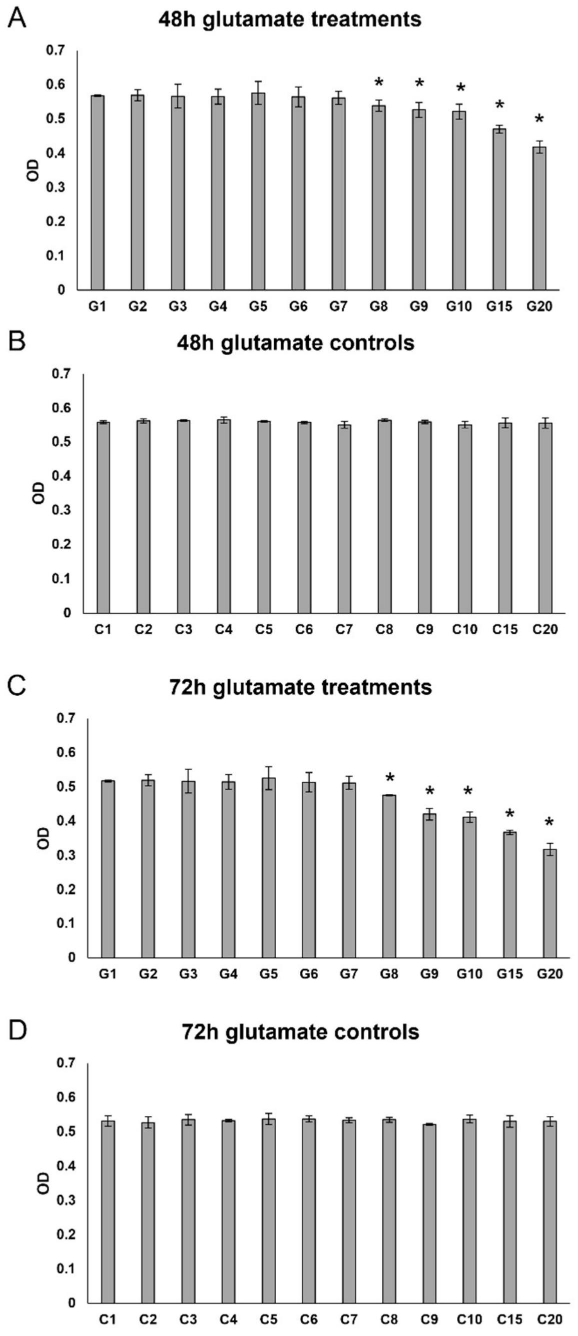

Supplementary Figure S3. The viability of cells after 48 h following treatment with **different concentrations of** glutamate (**A**) and the corresponding DMSO control (**B**); and after 72 h following treatment with different concentrations of glutamate (**C**) and the corresponding DMSO control (**D**). Significant differences ($p < 0.05$) compared to the DMSO controls are indicated by the * symbol. Abbreviations: G1-1mM glutamate; G2-2mM glutamate; G3-3mM glutamate; G4-4mM glutamate; G5-5mM glutamate; G6-6mM glutamate; G7-7mM glutamate; G8-8mM glutamate; G9-9mM glutamate; G10-10mM glutamate; G15-15mM glutamate; G20-20mM glutamate; C1-control of 1mM glutamate; C1-control of 1mM glutamate; C2-control of 2mM glutamate; C3-control of 3mM glutamate; C4-control of 4mM glutamate; C5-control of 5mM glutamate; C6-control of 6mM glutamate; C7-control of 7mM glutamate; C8-control of 8mM glutamate; C9-

control of 9mM glutamate; C10-control of 10mM glutamate; C15-control of 15mM glutamate; C20-control of 20mM glutamate (*n*=5).

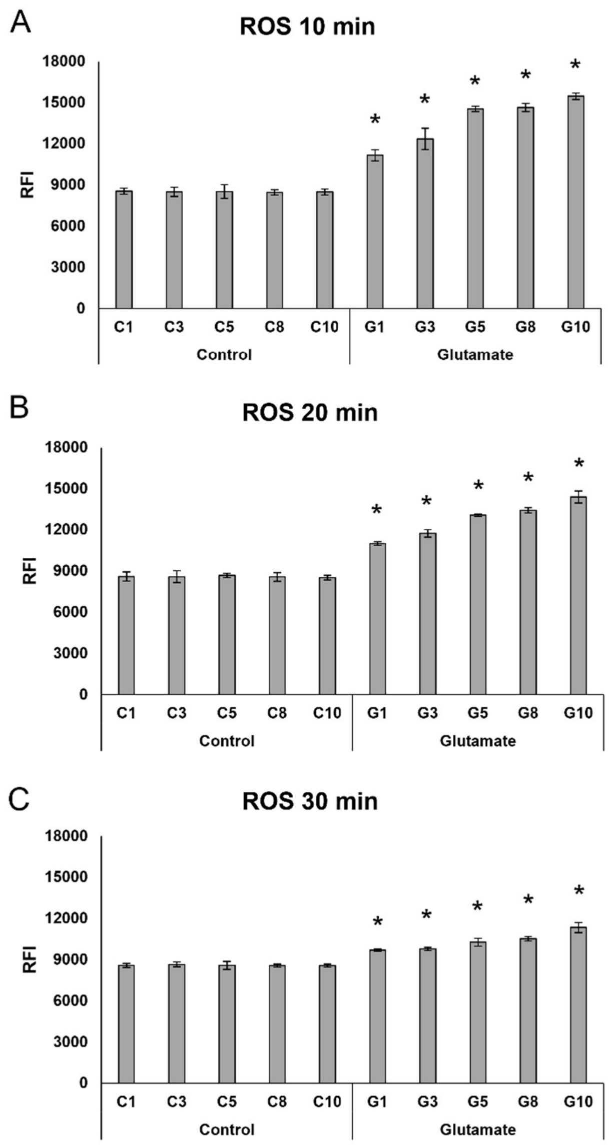

Supplementary Figure S4. Reactive Oxygen Species levels after 10 min (**A**), 20 min (**B**), and 30 min (**C**) of glutamate and its DMSO control treatments. Significant differences (p < 0.05) compared to the controls are indicated by the * symbol. Abbreviations: G1-1mM glutamate; G3-3mM glutamate; G5-5mM glutamate; G6-6mM glutamate; G8-8mM glutamate; G10-10mM glutamate; C1-control of 1mM glutamate; C1-control of 1mM glutamate; C3-control of 3mM glutamate; C5-control of 5mM glutamate; C8-control of 8mM glutamate; C10-control of 10mM glutamate (*n*=5).

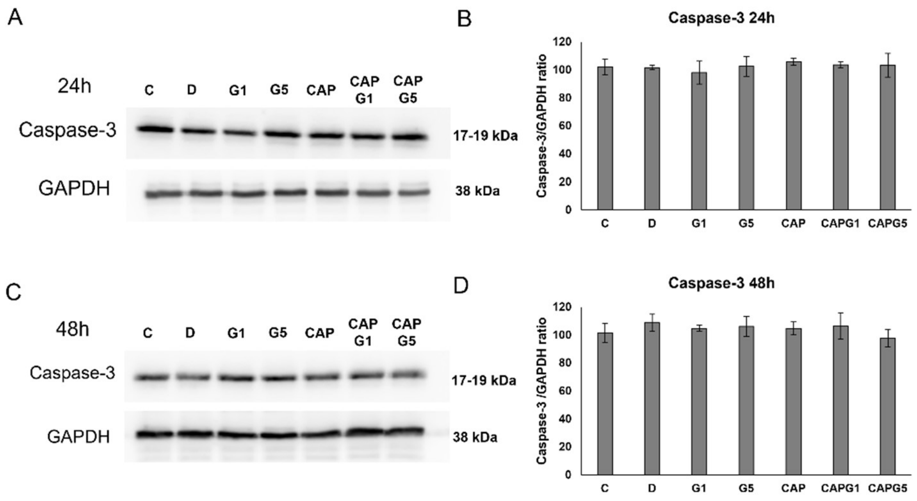

Supplementary Figure S5. The caspase-3 protein levels after 24h (**A, C**) and 48h (**B, D**) of capsanthin and glutamate treatment. Abbreviations: C-absolute control; D-DMSO control; G1-1mM glutamate; G5-5mM glutamate; CAP-capsanthin; CAPG1-capsanthin and 1mM glutamate; CAPG5-capsanthin and 5mM glutamate. (*n*=3)

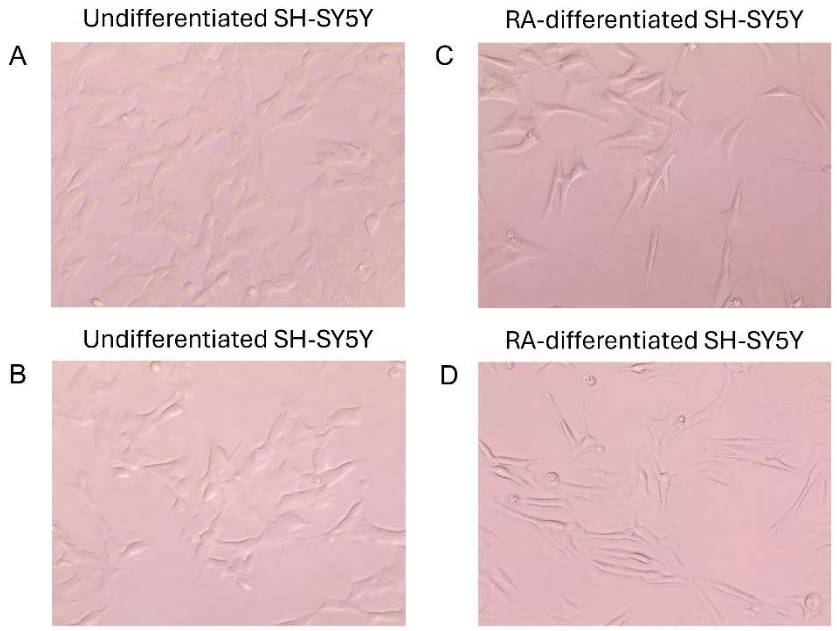

Supplementary Figure S6. Representative phase-contrast images of undifferentiated (**A,B**) and 5-day RA-differentiated (**C,D**) SH-SY5Y. Images were acquired using an EVOS XL Core microscope (Invitrogen, Carlsbad, CA, USA) with a Plan PH2 20×/0.40 objective.

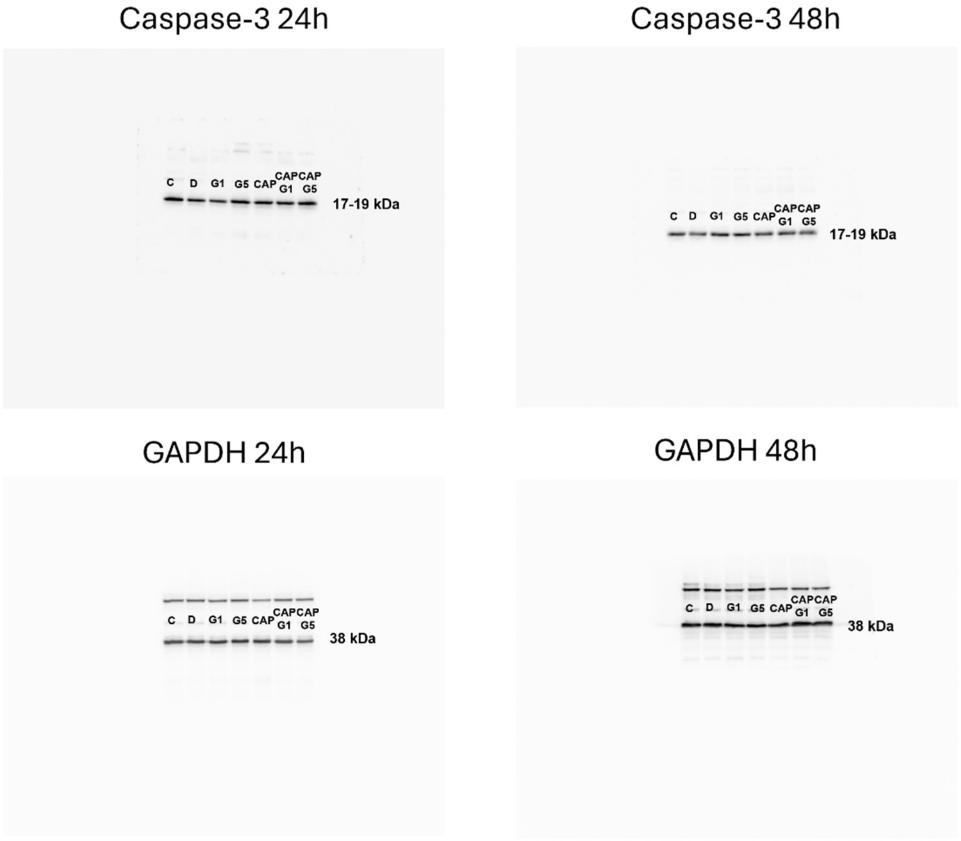

Supplementary Figure S7. Caspase-3 and GAPDH original western blot images with sample and labels. Abbreviations: C-absolute control; D-DMSO control; G1-1mM glutamate; G5-5mM glutamate; CAP-capsanthin; CAPG1-capsanthin and 1mM glutamate; CAPG5-capsanthin and 5mM glutamate.

A

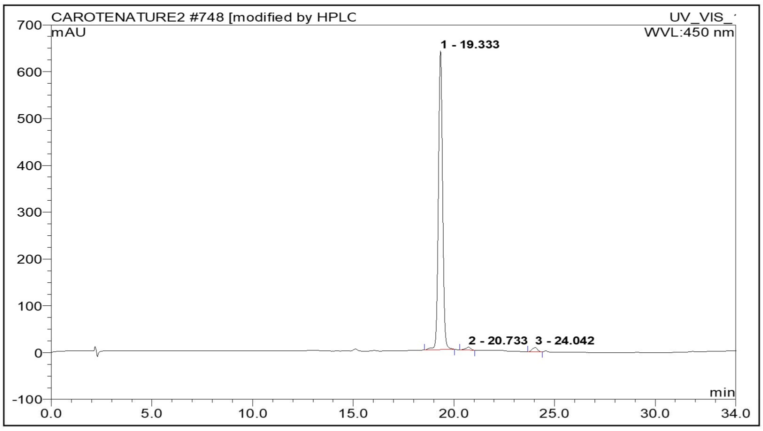

B

No.	Ret.Time	Peak Name	Height	Area	Rel.Area
	min		mAU	mAU*min	%
1	19,33	Capsantin	637,078	154,393	97,17
2	20,73	n.a.	6,106	1,820	1,15
3	24,04	n.a.	8,998	2,672	1,68
Total:			652,181	158,885	100,00

C

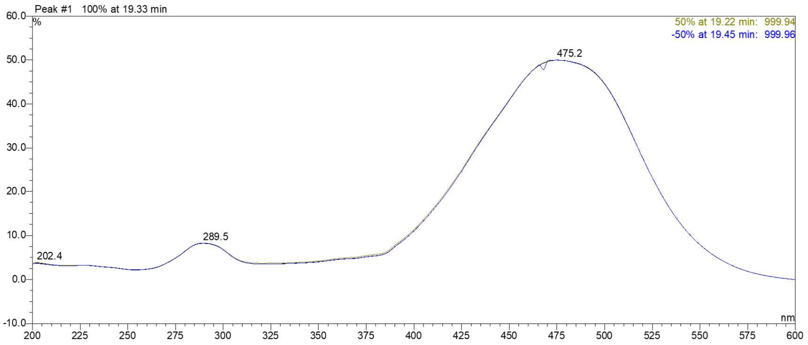

Supplementary Figure S8. (**A**) Analytical HPLC chromatogram recorded at 450 nm showing the major capsanthin peak and minor components. (**B**) Peak integration table with retention times and relative peak areas (%) used for purity estimation by peak area normalization. (**C**) UV–Vis spectrum of the main HPLC peak (capsanthin).
